# Supplementary material for: Proteostasis perturbation of N-Myc leveraging HSP70 mediated protein turnover improves treatment of neuroendocrine prostate cancer
Source: Nat Commun. 2024 Aug 5;15:6626. doi: 10.1038/s41467-024-50459-x (PMC11300456; doi:10.1038/s41467-024-50459-x)
Supplement: Supplementary file 1 — Supplementary Information [file 41467_2024_50459_MOESM1_ESM.pdf]

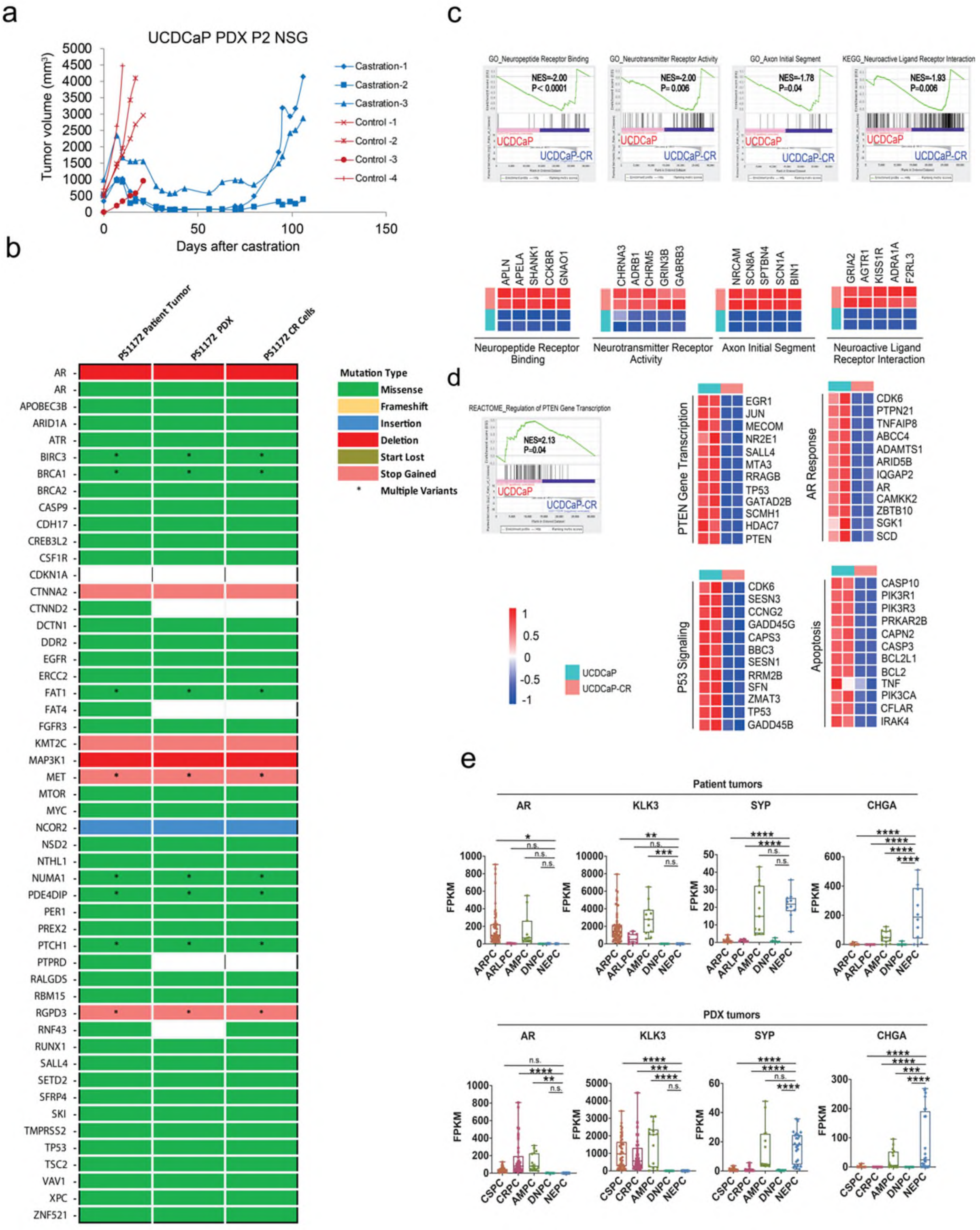

a
UCDCaP PDX P2 NSG
Tumor volume (mm³)
Days after castration
Castration-1
Castration-2
Castration-3
Control -1
Control -2
Control -3
Control -4
b
P51172 Patient Tumor
P51172 PDX
P51172 CR Cells
Mutation Type
Missense
Frameshift
Insertion
Deletion
Start Lost
Stop Gained
* Multiple Variants
AR
AR
APOBEC3B
ARID1A
ATR
BIRC3
BRCA1
BRCA2
CASP9
CDH17
CREB3L2
CSF1R
CDKN1A
CTNNA2
CTNND2
DCTN1
DDR2
EGFR
ERCC2
FAT1
FAT4
FGFR3
KMT2C
MAP3K1
MET
MTOR
MYC
NCOR2
NSD2
NTHL1
NUMA1
PDE4DIP
PER1
PREX2
PTCH1
PTPRD
RALGDS
RBM15
RGPD3
RNF43
RUNX1
SALL4
SETD2
SFRP4
SKI
TMPRSS2
TP53
TSC2
VAV1
XPC
ZNF521
c
GO_Neuropeptide Receptor Binding
NES=-2.00
P< 0.0001
GO_Neurotransmitter Receptor Activity
NES=-2.00
P= 0.006
GO_Axon Initial Segment
NES=-1.78
P=0.04
KEGG_Neuroactive Ligand Receptor Interaction
NES=-1.93
P=0.006
UCDCaP
UCDCaP-CR
APLN
APELA
SHANK1
CCKBR
GNAO1
CHRNA3
ADRB1
CHRM5
GRIN3B
GABRB3
NRCAM
SCN8A
SPTBN4
SCN1A
BIN1
GRIA2
AGTR1
KISS1R
ADRA1A
F2RL3
Neuropeptide Receptor Binding
Neurotransmitter Receptor Activity
Axon Initial Segment
Neuroactive Ligand Receptor Interaction
d
REACTOME_Regulation of PTEN Gene Transcription
NES=2.13
P=0.04
UCDCaP
UCDCaP-CR
PTEN Gene Transcription
EGR1
JUN
MECOM
NR2E1
SALL4
MTA3
RRAGB
TP53
GATAD2B
SCMH1
HDAC7
PTEN
AR Response
CDK6
PTPN21
TNFAIP8
ABCC4
ADAMTS1
ARID5B
IQGAP2
AR
CAMKK2
ZBTB10
SGK1
SCD
P53 Signaling
CDK6
SESN3
CCNG2
GADD45G
CAPS3
BBC3
SESN1
RRM2B
SFN
ZMAT3
TP53
GADD45B
Apoptosis
CASP10
PIK3R1
PIK3R3
PRKAR2B
CAPN2
CASP3
BCL2L1
BCL2
TNF
PIK3CA
CFLAR
IRAK4
e
Patient tumors
AR
KLK3
SYP
CHGA
FPKM
ARPC
ARLPC
AMPC
DNPC
NEPC
PDX tumors
CSPC
CRPC
AMPC
DNPC
NEPC
n.s.

Supplementary Fig. 1 Long-term androgen-deprivation patient derived prostate xenograft tumor exhibits neural lineage plasticity and overexpresses N-Myc. a Tumor growth curves of the second passage of UCDCaP patient derived xenograft (PDX) with (n=4) or without (n=3) castration over three months of period. **b** Representative mutations found in UCDCaP patient tumor, PDX, and cells. **c** Gene Set Enrichment Analysis (GSEA) of the gene signatures up or down regulated in UCDCaP-CR cells compared with UCDCaP cells. The signature was defined by genes that are preferentially upregulated in the neuropeptide receptor binding, neurotransmitter receptor activity, axon initial segment and neuroactive ligand receptor interaction pathways. **d** GSEA of the PTEN gene transcription signatures were downregulated in UCDCaP-CR. Heatmap showing the up-regulation or down-regulation of genes related to these signaling pathways, as well as the AR response, P53 signaling, apoptosis pathways. **e** The mRNA expression of AR, KLK3, SYP, and CHGA were analyzed in patients' samples (n=96) and PDX samples (n=145). Center: median; box: 25th to 75th interquartile range (IQR); whiskers: 1.5× IQR; outliers: individual data points; statistical significance determined by one-way ANOVA with a Tukey multiple-comparison test. AR-high prostate cancer (ARPC), AR-low prostate cancer (ARLPC), amphicrine prostate cancer (AMPC), double negative prostate cancer (DNPC), castration-sensitive prostate cancer (CSPC), castration-resistant prostate cancer (CRPC). *n.s. not significant;* $*p < 0.05$; $**p < 0.01$; $***p < 0.001$; *and* $****p < 0.0001$. Source data are provided as a Source Data file.

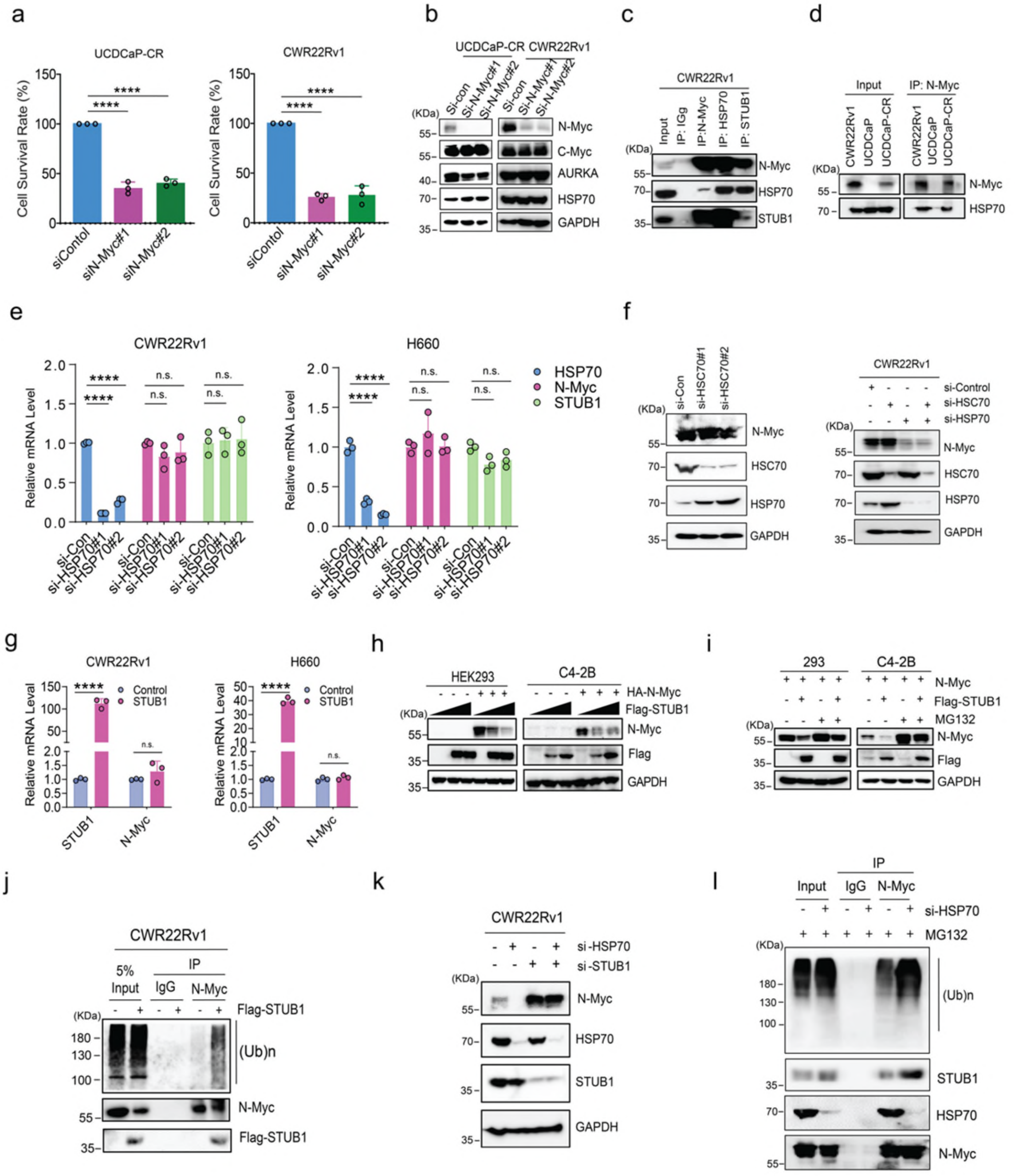
a
UCDCaP-CR
CWR22Rv1
Cell Survival Rate (%)
siContol
siN-Myc#1
siN-Myc#2
****
b
UCDCaP-CR
CWR22Rv1
Si-con
Si-N-Myc#1
Si-N-Myc#2
(KDa)
N-Myc
C-Myc
AURKA
HSP70
GAPDH
c
CWR22Rv1
Input
IP: IGg
IP:N-Myc
IP: HSP70
IP: STUB1
N-Myc
HSP70
STUB1
d
Input
IP: N-Myc
CWR22Rv1
UCDCaP
UCDCaP-CR
N-Myc
HSP70
e
CWR22Rv1
H660
Relative mRNA Level
HSP70
N-Myc
STUB1
si-Con
si-HSP70#1
si-HSP70#2
n.s.
f
si-Con
si-HSC70#1
si-HSC70#2
N-Myc
HSC70
HSP70
GAPDH
CWR22Rv1
si-Control
si-HSC70
si-HSP70
g
CWR22Rv1
H660
Relative mRNA Leval
Control
STUB1
N-Myc
h
HEK293
C4-2B
HA-N-Myc
Flag-STUB1
N-Myc
Flag
GAPDH
i
293
C4-2B
N-Myc
Flag-STUB1
MG132
Flag
GAPDH
j
CWR22Rv1
5% Input
IP
IgG
N-Myc
Flag-STUB1
(Ub)n
k
CWR22Rv1
si-HSP70
si-STUB1
N-Myc
HSP70
STUB1
GAPDH
l
IP
Input
IgG
N-Myc
si-HSP70
MG132
(Ub)n
STUB1
HSP70
N-Myc

Supplementary Fig. 2 HSP70/STUB1 controls N-Myc protein levels through ubiquitin proteasome system. a, b UCDCaP-CR and CWR22Rv1 cells were transfected with siRNA against N-Myc or negative control for 7 days. The cell proliferation (**a**, n = 3 samples) and N-Myc, c-Myc, AURKA, HSP70, and GAPDH protein expression (**b**) were assessed. **c** The whole cell lysates from CWR22Rv1 cells were immunoprecipitated with anti-N-Myc, anti-HSP70, anti-STUB1, or anti-IgG antibodies and probed for N-Myc, HSP70 and STUB1, respectively. **d** The whole cell lysates from CWR22Rv1, UCDCaP, and UCDCaP-CR cells were immunoprecipitated with anti-N-Myc antibody and probed for N-Myc and HSP70. **e** The mRNA expression of HSP70, N-Myc, and STUB1 was assessed in H660 and CWR22Rv1 cells transfected with siRNA against HSP70 or negative control for 7 days (n = 3 samples). **f** CWR22Rv1 cells were transfected with siHSC70 #1 and #2 (left) for 3 days, and the levels of N-Myc, HSC70, and HSP70 were examined by western blotting with GAPDH as the internal control. CWR22Rv1 cells were transfected with siHSC70, siHSP70, or both (right) for 3 days and the levels of N-Myc, HSC70, and HSP70 were assessed. **g** The mRNA expression of STUB1 and N-Myc was determined after H660 and CWR22Rv1 cells transfected with 4μg of Flag-STUB1 plasmids for 3 days (n = 3 samples). **h** HEK293 and C4-2B cells were transfected with HA-N-Myc with or without 0, 2μg, or 4μg Flag-STUB1 plasmids for 3 days. The protein expression of N-Myc was determined. **i,** HEK293 and C4-2B cells were co-transfected with HA-N-Myc and Flag-STUB1 plasmids for 3 days and then treated with or without MG132 (5 μM) for 6 hours. The expression of N-Myc was determined by western blotting. **j** CWR22Rv1 cells were transfected with Flag-STUB1 plasmids for 3 days. Immunoprecipitation was performed with an anti-N-Myc antibody and probed for ubiquitin, N-Myc, and Flag-STUB1, respectively. **k** CWR22Rv1 cells were treated with siHSP70, siSTUB1 or both and the expressions of N-Myc, HSP70 and STUB1 were determined by western blots. **l** CWR22Rv1 cells were transfected with siHSP70 for 3 days, and then treated with 5 μM MG132 overnight. Immunoprecipitation was performed with an anti-N-Myc antibody and probed for ubiquitin, STUB1, HSP70 and N-Myc, respectively. Results are the mean of three independent experiments (± S.D.). For **a** and **e**, statistical significance determined by one-way ANOVA with a Tukey multiple-comparison test. For **g**, statistical significance determined by unpaired two-sided t-test. Results are the mean of three independent experiments (± S.D.). *n.s. not significant;* ****$p < 0.0001$. Source data are provided as a Source Data file.

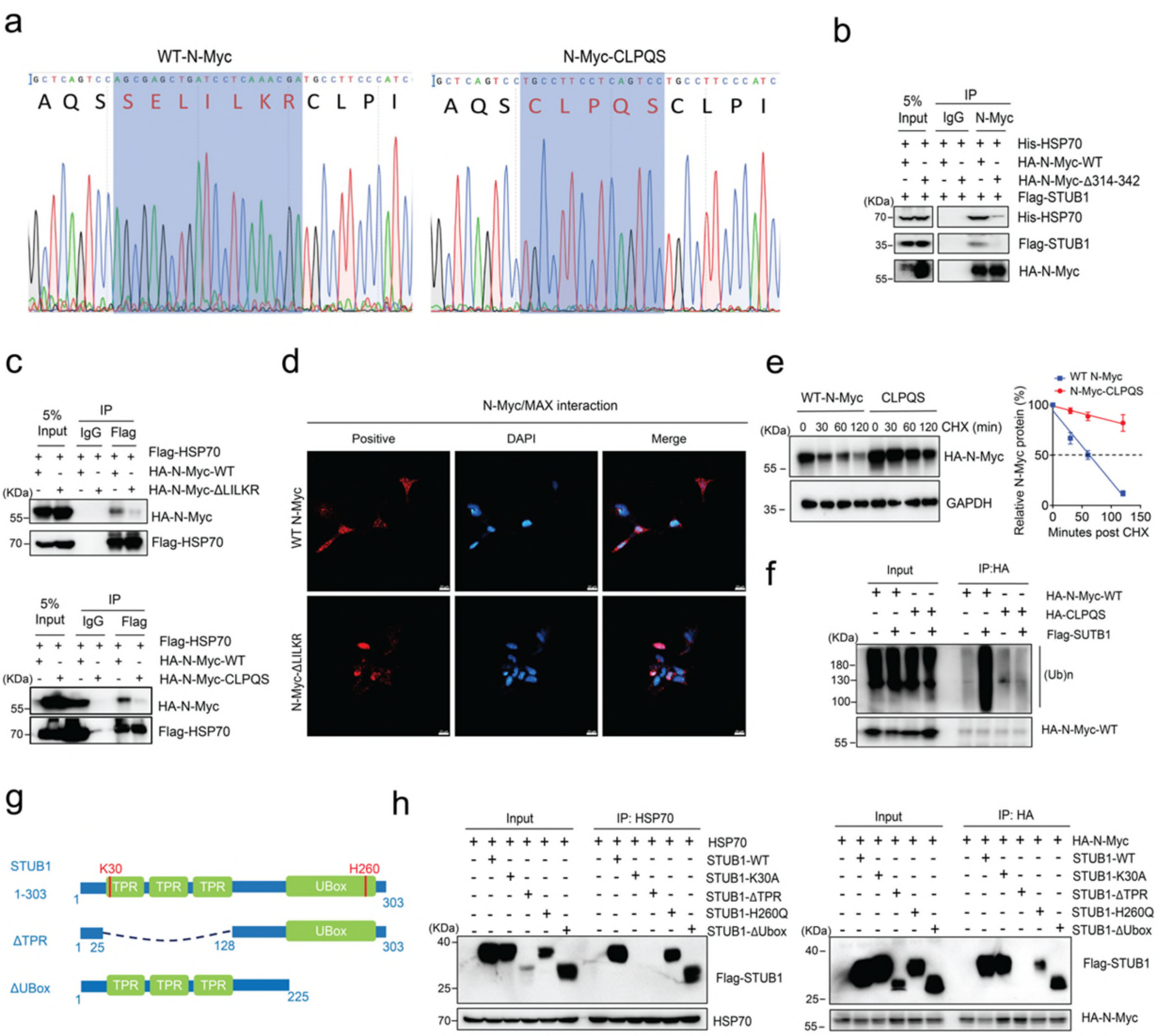

a
WT-N-Myc
A Q S S E L I L K R C L P I
N-Myc-CLPQS
A Q S C L P Q S C L P I
b
5% Input
IP
IgG
N-Myc
His-HSP70
HA-N-Myc-WT
HA-N-Myc-Δ314-342
Flag-STUB1
(KDa)
70
35
55
His-HSP70
Flag-STUB1
HA-N-Myc
c
5% Input
IP
IgG
Flag
Flag-HSP70
HA-N-Myc-WT
HA-N-Myc-ΔLILKR
(KDa)
55
70
HA-N-Myc
Flag-HSP70
HA-N-Myc-CLPQS
d
N-Myc/MAX interaction
Positive
DAPI
Merge
WT N-Myc
N-Myc-ΔLILKR
e
WT-N-Myc
CLPQS
(KDa)
0 30 60 120 0 30 60 120 CHX (min)
55
35
HA-N-Myc
GAPDH
WT N-Myc
N-Myc-CLPQS
Relative N-Myc protein (%)
100
50
0
0 50 100 150
Minutes post CHX
f
Input
IP:HA
HA-N-Myc-WT
HA-CLPQS
Flag-SUTB1
(KDa)
180
130
100
55
(Ub)n
HA-N-Myc-WT
g
STUB1
1-303
K30
H260
TPR
TPR
TPR
UBox
1
303
ΔTPR
1 25
128
303
ΔUBox
1
225
h
Input
IP: HSP70
HSP70
STUB1-WT
STUB1-K30A
STUB1-ΔTPR
STUB1-H260Q
STUB1-ΔUbox
(KDa)
40
25
70
Flag-STUB1
HSP70
IP: HA
HA-N-Myc
55
Flag-STUB1
HA-N-Myc

Supplementary Fig. 3 HSP70 and STUB1 affects and directly interacts with N-Myc domain. **a** The Sanger Sequence Chromatogram of wild-type (WT) N-Myc and N-Myc-CLPQS plasmids. **b** HEK293 cells were co-transfected with His-HSP70 and Flag-STUB1 with or without HA-WT-N-Myc or HA-N-Myc-Δ314-342. Whole cell lysates were immunoprecipitated with anti-N-Myc antibody and probed for His-HSP70, Flag-STUB1 and HA-N-Myc, respectively. IgG antibodies were used as the negative control and whole lysate input were loaded alongside. **c** HEK293 cells were co-transfected with Flag-HSP70, HA-WT-N-Myc, HA-N-Myc-ΔLILKR, or HA-N-Myc-CLPQS. Whole cell lysates were immunoprecipitated with anti-Flag antibodies and probed for Flag-HSP70 and HA-N-Myc, respectively. **d** HEK293 cells were co-transfected with WT-N-Myc or N-Myc-ΔLILKR, with Max for 3 days, and the interaction of wild type and mutant N-Myc and Max was determined by PLA. Scale bar represents 20 microns. **e** HEK293 cells were transfected with HA-WT-N-Myc or HA-N-Myc-CLPQS and treated with 50 μg/ml cycloheximide for 0, 30, 60, and 120 minutes. Whole cell lysates were separated by electrophoresis and blotted with HA, and the half-life of N-Myc was calculated ($n = 3$ independent experiments and data are presented as mean ± S.D.). **f** HEK293 cells were co-transfected with HA-WT-N-Myc, HA-N-Myc-CLPQS, and Flag-STUB1. Whole cell lysates were immunoprecipitated with anti-HA antibodies and probed for ubiquitin and HA-N-Myc. **g** Schematic representations of STUB1 point mutations or deletion mutants used for the domain mapping. **h** HEK293 cells were co-transfected with Flag-WT-STUB1 or mutants with HSP70 or HA-N-Myc. Whole cell lysates were immunoprecipitated with the anti-HSP70 or anti-HA antibodies and immunoblotted with the Flag, HSP70, or HA antibodies. Source data are provided as a Source Data file.

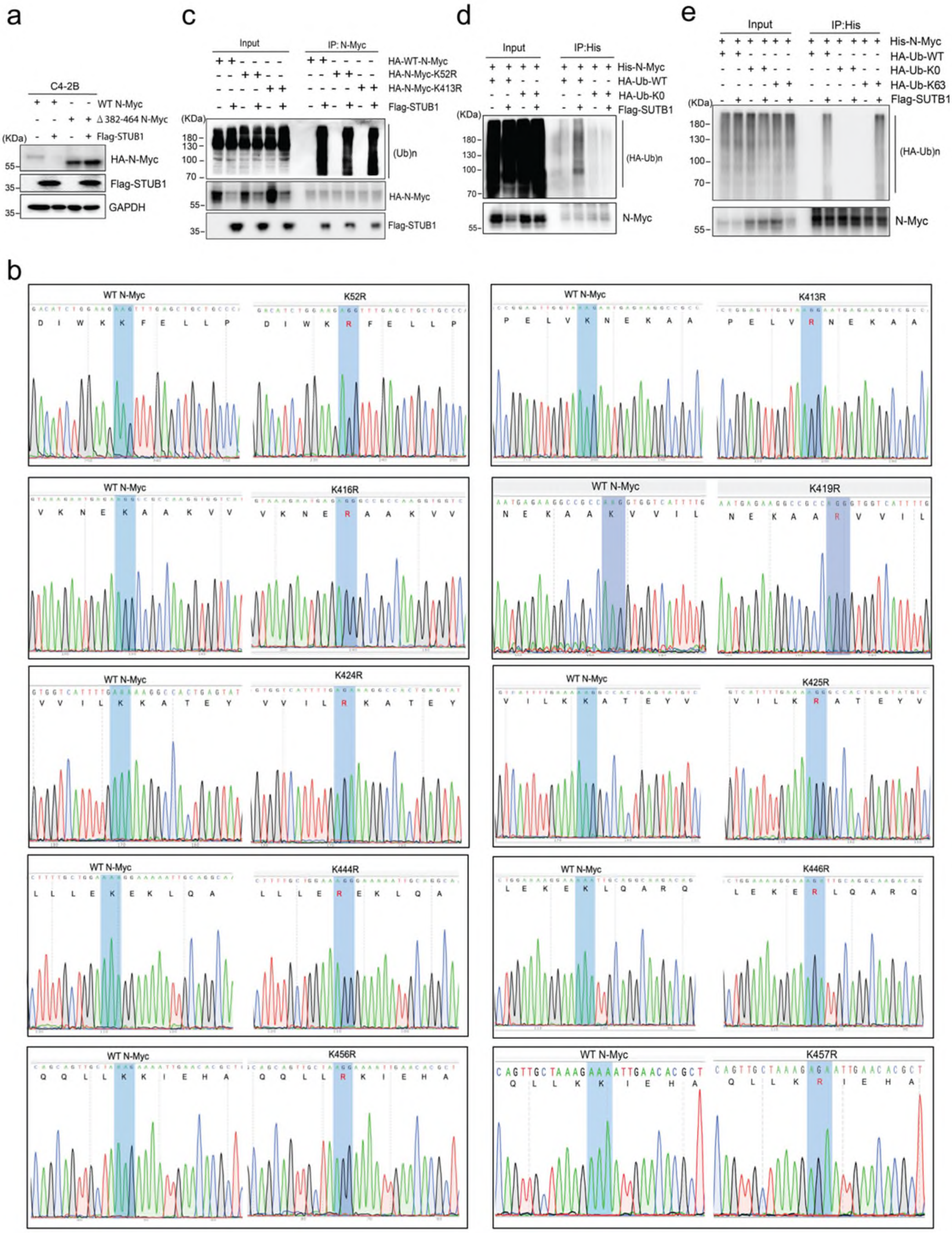
a
C4-2B
WT N-Myc
Δ 382-464 N-Myc
Flag-STUB1
(KDa)
55
35
35
HA-N-Myc
Flag-STUB1
GAPDH
c
Input
IP: N-Myc
HA-WT-N-Myc
HA-N-Myc-K52R
HA-N-Myc-K413R
Flag-STUB1
(KDa)
180
130
100
70
55
35
(Ub)n
HA-N-Myc
Flag-STUB1
d
Input
IP:His
His-N-Myc
HA-Ub-WT
HA-Ub-K0
Flag-SUTB1
(KDa)
180
130
100
70
55
(HA-Ub)n
N-Myc
e
Input
IP:His
His-N-Myc
HA-Ub-WT
HA-Ub-K0
HA-Ub-K63
Flag-SUTB1
(KDa)
180
130
100
70
55
(HA-Ub)n
N-Myc
b
WT N-Myc
D I W K K F E L L P
K52R
D I W K R F E L L P
WT N-Myc
P E L V K N E K A A
K413R
P E L V R N E K A A
WT N-Myc
V K N E K A A K V V
K416R
V K N E R A A K V V
WT N-Myc
N E K A A K V V I L
K419R
N E K A A R V V I L
WT N-Myc
V V I L K K A T E Y
K424R
V V I L R K A T E Y
WT N-Myc
V I L K K A T E Y V
K425R
V I L K R A T E Y V
WT N-Myc
L L L E K E K L Q A
K444R
L L L E R E K L Q A
WT N-Myc
L E K E K L Q A R Q
K446R
L E K E R L Q A R Q
WT N-Myc
Q Q L L K K I E H A
K456R
Q Q L L R K I E H A
WT N-Myc
CAGTTGCTAAAGAAAATTGAACACGCT
Q L L K K I E H A
K457R
CAGTTGCTAAAGAGAATTGAACACGCT
Q L L K R I E H A

Supplementary Fig. 4 STUB1 coordinates with HSP70 in regulating N-Myc polyubiquitination and degradation. a C4-2B cells were co-transfected with HA-WT-N-Myc or △382-464-N-Myc with or without Flag-STUB1. The expression of N-Myc was determined by western blotting. **b** The Sanger Sequence chromatogram of wild-type (WT) N-Myc and eight mutant plasmids (K52R, K413R, K416R, K419R K424R, K425R, K444R, K446R, K456R, and K457R). **c** HEK293 cells were co-transfected with HA-WT-N-Myc or mutants (K52R, K413R) with and without Flag-STUB1. Whole cell lysates were immunoprecipitated with anti-N-Myc antibodies and blotted with ubiquitin, Flag, or HA antibodies. **d** HEK293 cells were co-transfected with His-N-Myc, HA-WT-Ubiquitin or HA-Ubiquitin-K0 and Flag-STUB1. The cell lysates were immunoprecipitated with the anti-His antibody and blotted with anti-HA or N-Myc antibodies. **e** HEK293 cells were co-transfected with His-N-Myc, HA-WT-Ubiquitin or HA-Ubiquitin-K0 or HA-Ubiquitin-K63 with and without Flag-STUB1. Whole cell lysates were immunoprecipitated with anti-His antibodies and blotted with HA and N-Myc antibodies. Source data are provided as a Source Data file.

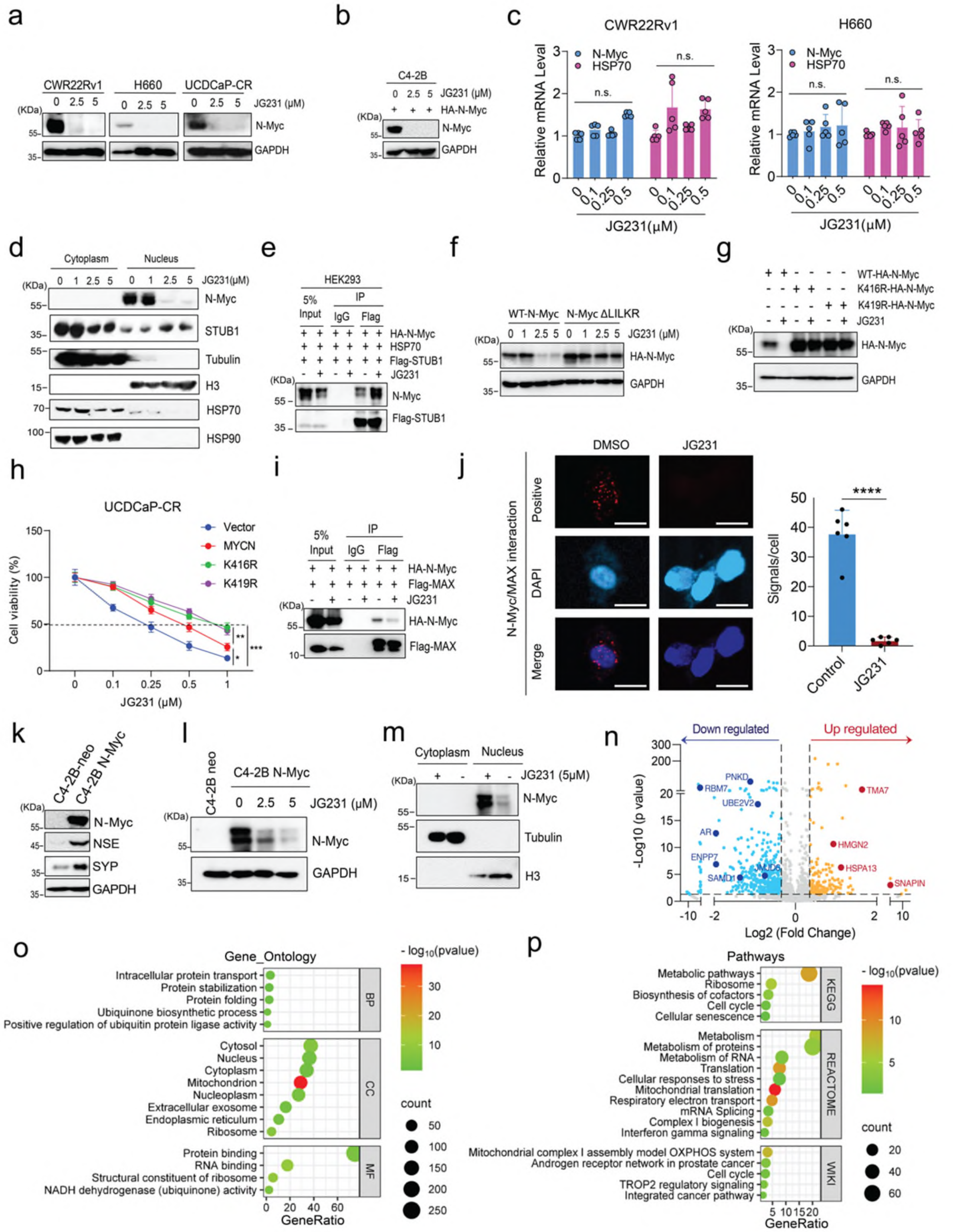

a
CWR22Rv1
H660
UCDCaP-CR
0 2.5 5 0 2.5 5 0 2.5 5 JG231 (μM)
(KDa)
N-Myc
GAPDH
b
C4-2B
0 2.5 5 JG231 (μM)
+ + + HA-N-Myc
N-Myc
GAPDH
c
CWR22Rv1
H660
N-Myc
HSP70
Relative mRNA Leval
n.s.
JG231(μM)
d
Cytoplasm
Nucleus
0 1 2.5 5 0 1 2.5 5 JG231(μM)
N-Myc
STUB1
Tubulin
H3
HSP70
HSP90
e
HEK293
5% Input
IP
IgG
Flag
HA-N-Myc
HSP70
Flag-STUB1
JG231
N-Myc
Flag-STUB1
f
WT-N-Myc
N-Myc ΔLILKR
0 1 2.5 5 0 1 2.5 5 JG231 (μM)
HA-N-Myc
GAPDH
g
WT-HA-N-Myc
K416R-HA-N-Myc
K419R-HA-N-Myc
JG231
HA-N-Myc
GAPDH
h
UCDCaP-CR
Vector
MYCN
K416R
K419R
Cell viability (%)
JG231 (μM)
i
5% Input
IP
IgG
Flag
HA-N-Myc
Flag-MAX
JG231
HA-N-Myc
Flag-MAX
j
DMSO
JG231
N-Myc/MAX interaction
Positive
DAPI
Merge
Signals/cell
****
Control
JG231
k
C4-2B-neo
C4-2B N-Myc
N-Myc
NSE
SYP
GAPDH
l
C4-2B neo
C4-2B N-Myc
0 2.5 5 JG231 (μM)
N-Myc
GAPDH
m
Cytoplasm
Nucleus
JG231 (5μM)
N-Myc
Tubulin
H3
n
Down regulated
Up regulated
PNKD
RBM7
UBE2V2
AR
ENPP7
SAMD1
JMJD6
TMA7
HMGN2
HSPA13
SNAPIN
-Log10 (p value)
Log2 (Fold Change)
o
Gene_Ontology
Intracellular protein transport
Protein stabilization
Protein folding
Ubiquinone biosynthetic process
Positive regulation of ubiquitin protein ligase activity
Cytosol
Nucleus
Cytoplasm
Mitochondrion
Nucleoplasm
Extracellular exosome
Endoplasmic reticulum
Ribosome
Protein binding
RNA binding
Structural constituent of ribosome
NADH dehydrogenase (ubiquinone) activity
BP
CC
MF
GeneRatio
- log10(pvalue)
count
p
Pathways
Metabolic pathways
Ribosome
Biosynthesis of cofactors
Cell cycle
Cellular senescence
Metabolism
Metabolism of proteins
Metabolism of RNA
Translation
Cellular responses to stress
Mitochondrial translation
Respiratory electron transport
mRNA Splicing
Complex I biogenesis
Interferon gamma signaling
Mitochondrial complex I assembly model OXPHOS system
Androgen receptor network in prostate cancer
Cell cycle
TROP2 regulatory signaling
Integrated cancer pathway
KEGG
REACTOME
WIKI
GeneRatio
- log10(pvalue)
count

Supplementary Fig. 5 Pharmacologically targeting HSP70 degrades N-Myc expression *via* STUB1. a CWR22Rv1, H660, and UCDCaP-CR cells were treated with JG231 (0, 2.5, or 5 μM) for 24 hours and whole cell lysates were separated by electrophoresis and probed for N-Myc and GAPDH antibodies. **b** C4-2B cells were transfected with HA-N-Myc plasmids for 2 days and then cells were treated with JG231 (0, 2.5, or 5 μM) for 24 hours. Whole cell lysates were collected and subjected to western blotting (left). **c** CWR22Rv1 and H660 cells were treated with JG231 (0, 0.1, 0.25, or 0.5 μM) for 3 days. The mRNA expression of N-Myc and HSP70 was determined by RT-PCR (n = 5 samples). **d** Western blotting results showed the cytoplasmic and nuclear expression of N-Myc, STUB1, HSP70, and HSP90 in CWR22Rv1 cells treated with or without JG231 (1, 2.5, and 5 μM) for 24 hours. Tubulin and Histone-H3 were used as the loading controls for cytoplasm and nuclei, respectively. **e** HEK293 cells were co-transfected with HA-N-Myc, HSP70 and Flag-STUB1 expression constructs for 3 days, then cells were treated with JG231 (2.5 μM) for 24 hours. Whole cell lysates were harvested and immunoprecipitated with the Flag antibody and blotted with anti-Flag, and anti-N-Myc antibodies. **f** HEK293 cells were transfected with WT-N-Myc or N-Myc- ΔLILKR plasmids for 3 days, and then treated with JG231 (0, 1, 2.5 and 5 μM) for 24 hours. N-Myc expression was determined by western blotting. **g** HEK293 cells were transfected with WT-N-Myc, N-Myc-K416R, or N-Myc-K419R plasmids for 3 days, and then treated with JG231 (2.5 μM) for 24 hours. N-Myc expression was determined by western blotting. **h** UCDCaP-CR cells were transfected with vector, WT-MYCN, K416R-MYCN, or K419R-MYCN for 24 hours, following treatment with different doses of JG231 for 3 days, the cell viability was determined (n = 3 samples). **i** HEK293 cells were co-transfected with HA-N-Myc and Flag-MAX plasmids for 3 days, and then treated with JG231 (2.5 μM) for 24 hours. Western blotting was performed after cell lysates were precipitated with anti-Flag or IgG antibodies and probed for the detection of HA-N-Myc and Flag-MAX. **j** The PLA for N-Myc and MAX interaction after treatment of CWR22Rv1 cells with JG231 (2.5 μM) for 24 hours. Red signals represent the proximity of the N-Myc and MAX proteins. DNA is stained with DAPI. Quantification of the PLA signals per cell after treatment with the different compounds (n = 6). **k** The protein expressions of N-Myc, NSE, and SYP in C4-2B neo and C4-2B N-Myc cells were determined by western blotting. **l** C4-2B N-Myc cells were treated JG231 (0, 2.5, and 5 μM) for 4 hours, the expression of N-Myc was determined. C4-2B neo was set up as negative control. **m** Western blotting results showed the cytoplasmic and nuclear expression of N-Myc in C4-2B N-Myc cells treated with or without JG231 (0 and 5 μM) for 4 hours. Tubulin and Histone-H3 were used as the loading controls for cytoplasm and nuclei, respectively. **n** Volcano plot displaying up-regulated

proteins (in yellow and red) and down-regulated proteins (in blue) from whole cell lysates following JG231 treatment in C4-2B N-Myc cells, in comparison with DMSO treatment. **o, p** Gene Ontology and pathway analyses demonstrate the enrichment of functional annotations in down-regulated proteins in whole-cell lysates from C4-2B N-Myc cells treated with 5 μM JG231 for 4 hours. Results are the mean of three independent experiments (± S.D.). For **c**, statistical significance determined by one-way ANOVA with a Tukey multiple-comparison test. For **h** and **j**, statistical significance determined by unpaired two-sided t-test. *$p < 0.05$; **$p < 0.01$; ***$p < 0.001$; *n.s. not significant*. Source data are provided as a Source Data file.

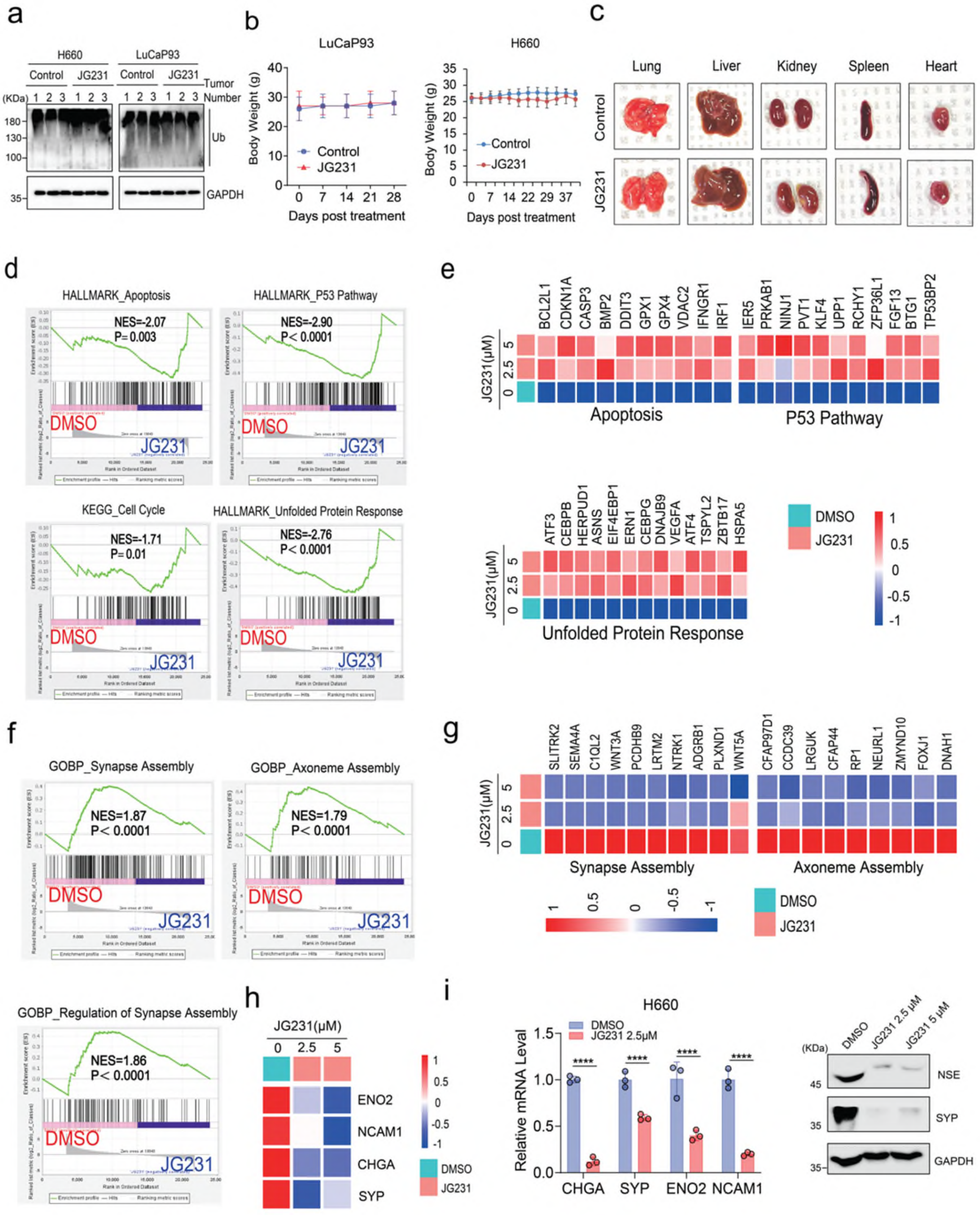
a
H660
LuCaP93
Control
JG231
Tumor Number
(KDa)
1 2 3 1 2 3
180
130
100
35
Ub
GAPDH
b
LuCaP93
H660
Body Weight (g)
Control
JG231
Days post treatment
c
Lung
Liver
Kidney
Spleen
Heart
Control
JG231
d
HALLMARK_Apoptosis
NES=-2.07
P= 0.003
HALLMARK_P53 Pathway
NES=-2.90
P< 0.0001
KEGG_Cell Cycle
NES=-1.71
P= 0.01
HALLMARK_Unfolded Protein Response
NES=-2.76
P< 0.0001
DMSO
JG231
e
BCL2L1
CDKN1A
CASP3
BMP2
DDIT3
GPX1
GPX4
VDAC2
IFNGR1
IRF1
IER5
PRKAB1
NINJ1
PVT1
KLF4
UPP1
RCHY1
ZFP36L1
FGF13
BTG1
TP53BP2
JG231(μM)
Apoptosis
P53 Pathway
ATF3
CEBPB
HERPUD1
ASNS
EIF4EBP1
ERN1
CEBPG
DNAJB9
VEGFA
ATF4
TSPYL2
ZBTB17
HSPA5
Unfolded Protein Response
DMSO
JG231
f
GOBP_Synapse Assembly
NES=1.87
P< 0.0001
GOBP_Axoneme Assembly
NES=1.79
P< 0.0001
GOBP_Regulation of Synapse Assembly
NES=1.86
P< 0.0001
DMSO
JG231
g
SLITRK2
SEMA4A
C1QL2
WNT3A
PCDHB9
LRTM2
NTRK1
ADGRB1
PLXND1
WNT5A
CFAP97D1
CCDC39
LRGUK
CFAP44
RP1
NEURL1
ZMYND10
FOXJ1
DNAH1
JG231(μM)
Synapse Assembly
Axoneme Assembly
DMSO
JG231
h
JG231(μM)
0 2.5 5
ENO2
NCAM1
CHGA
SYP
DMSO
JG231
i
H660
DMSO
JG231 2.5μM
Relative mRNA Leval
****
CHGA
SYP
ENO2
NCAM1
(KDa)
DMSO
JG231 2.5 μM
JG231 5 μM
NSE
SYP
GAPDH
45
35
35

Supplementary Fig. 6 HSP70 inhibitor JG231 suppresses NEPC xenograft tumor growth. **a** Western blot of representative tumors from LuCaP93 and H660 models treated with or without JG231 probed by anti-Ub antibodies. **b** Mice body weight of control and JG231 treated groups in LuCaP93 (n = 9) and H660 models (n = 6). **c** Representative mouse organ morphology pictures of control and JG231 treated groups in LuCaP93 model. **d** GSEA of the apoptosis, P53 pathway, cell cycle, and unfolded protein response pathways were upregulated in H660 cells treated with JG231. **e** Heatmap showing genes for the apoptosis, P53 pathway, cell cycle, and unfolded protein response were upregulated in H660 cells after JG231 treatment. **f** GSEA of the synapse assembly, axoneme assembly, and regulation of synapse assembly pathways were downregulated in H660 cells treated with JG231. **g** Heatmap showing both genes for the synapse assembly and axoneme assembly were downregulated in H660 cells after JG231 treatment. **h** Heatmap showing genes for the downregulated neuroendocrine (NE) features in H660 cells treated with JG231. **i** The mRNA expression of CHGA, SYP, ENO2, and NCAM1 was assessed in H660 cells treated with JG231 (2.5 μM) for 24 hours (left, n = 3 samples, statistical significance determined by unpaired two-sided t-test). The protein expression of SYP and ENO2 was assessed in H660 cells treated with JG231 (0, 2.5, and 5μM) for 24 hours (right). Results are the mean of three independent experiments (± S.D.). ****$p < 0.0001$. Source data are provided as a Source Data file.

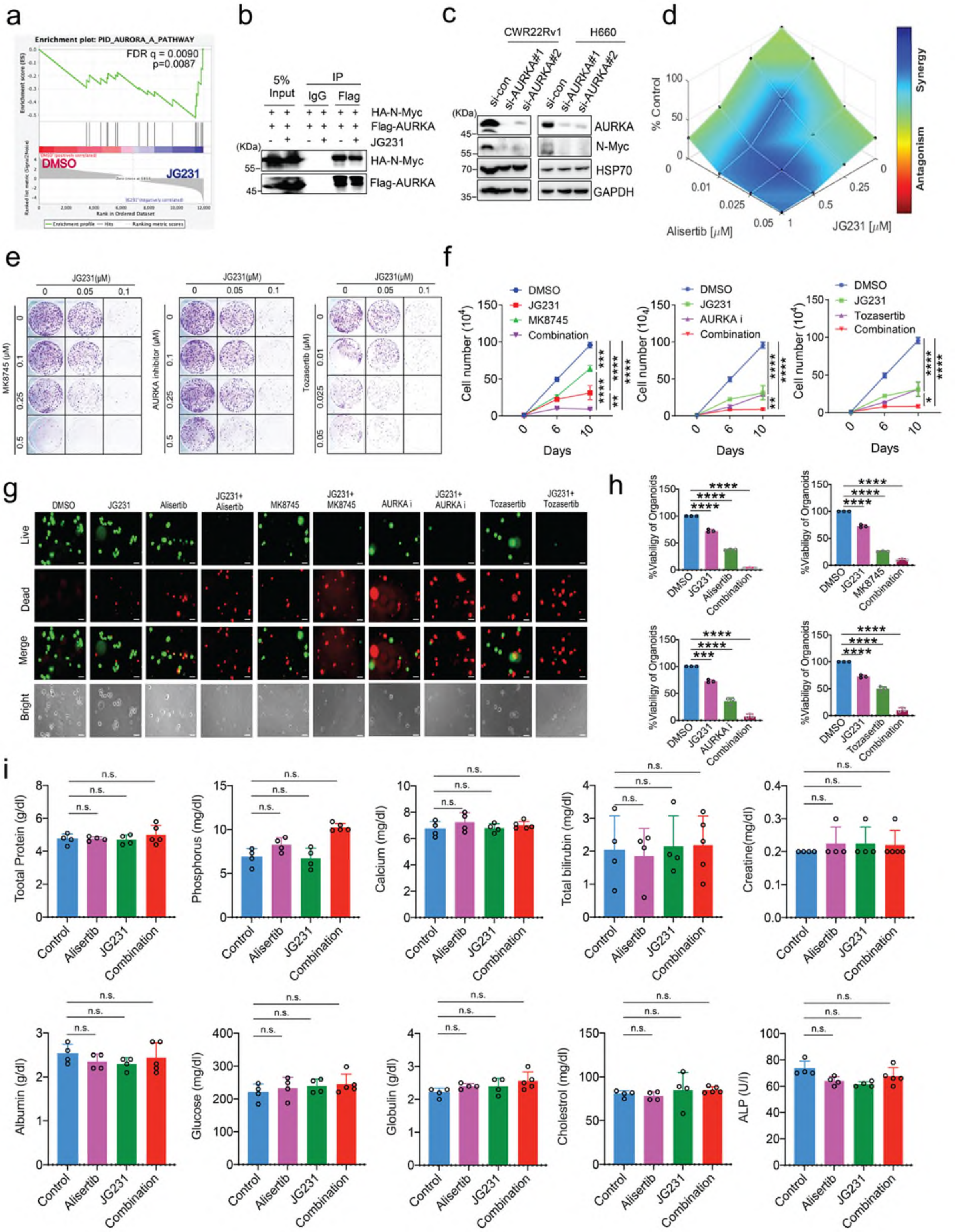
a
Enrichment plot: PID_AURORA_A_PATHWAY
FDR q = 0.0090
p=0.0087
DMSO
JG231
b
5% Input
IP
IgG
Flag
HA-N-Myc
Flag-AURKA
JG231
(KDa)
HA-N-Myc
Flag-AURKA
c
CWR22Rv1
H660
si-con
si-AURKA#1
si-AURKA#2
AURKA
N-Myc
HSP70
GAPDH
d
% Control
Synergy
Antagonism
Alisertib [μM]
JG231 [μM]
e
JG231(μM)
MK8745 (μM)
AURKA inhibitor (μM)
Tozasertib (μM)
f
DMSO
JG231
MK8745
Combination
AURKA i
Tozasertib
Cell number (10^4)
Days
g
DMSO
JG231
Alisertib
JG231+ Alisertib
MK8745
JG231+ MK8745
AURKA i
JG231+ AURKA i
Tozasertib
JG231+ Tozasertib
Live
Dead
Merge
Bright
h
%Vabiligy of Organoids
DMSO
JG231
Alisertib
MK8745
AURKA i
Tozasertib
Combination
i
n.s.
Control
Alisertib
JG231
Combination
Tootal Protein (g/dl)
Phosphorus (mg/dl)
Calcium (mg/dl)
Total bilirubin (mg/dl)
Creatine(mg/dl)
Albumin (g/dl)
Glucose (mg/dl)
Globulin (g/dl)
Cholestrol (mg/dl)
ALP (U/l)

Supplementary Fig. 7 Dual targeting of HSP70 and AURKA enhances N-Myc degradation and improves treatment in NEPC. a Gene Set Enrichment Analysis (GSEA) showing the enrichment of AURKA pathway in JG231 treated H660 cells. **b** HEK293 cells were co-transfected with HA-N-Myc and Flag-AURKA for 3 days, and then treated with JG231 (2.5 μM) for 24 hours. Whole cell lysates were immunoprecipitated with anti-Flag or anti-IgG antibodies and probed for HA-N-Myc and Flag-AURKA, respectively. **c** CWR22Rv1 and H660 cells were transfected with siRNA against AURKA or negative control for 5 days. N-Myc, AURKA, and HSP70 protein expression were assessed. **d** CWR22Rv1 cells were treated with different concentrations of JG231 with or without different concentrations of alisertib for 3 days. The drug synergy distribution map was generated by Combenefit. **e** CWR22Rv1 cells were treated with JG231 alone (0.05 and 0.1 μM) with or without MK8745 (0.1, 0.25, and 0.5μM), AURKA inhibitor (0.1, 0.25, and 0.5μM), and tozasertib (0.01, 0.025, and 0.05μM) for two weeks. The colony pictures in each condition were captured. **f** H660 cells were treated with MK8745 (0.5μM), AURKA inhibitor (0.5μM), Tozasertib (0.5μM), JG231 (0.1μM) or the combination for 6-10 days, the cell numbers were counted to determine the cell proliferation (n = 3 samples). **g, h** Organoids generated from H660 patient derived xenograft (PDX) tumors were treated with JG231 alone (0.1μM) with or without alisertib (0.5μM), MK-8745 (0.5μM), AURKA inhibitor (0.5μM), and tozasertib (0.5μM) for 7 days. Cell viability was determined by CellTiter-Glo Luminescent assay (n = 3 samples) and the live-and-dead cells were visualized by immunofluorescence. **i** Drug toxicity tests were conducted by measuring the total protein, phosphorus, calcium, total bilirubin, creatine, albumin glucose, globulin, cholesterol, and alkaline phosphatase (ALP) levels in the serum samples collected from animals treated with alisertib, JG231, combination, or the control (n = 4-5 samples). Results are the mean of three independent experiments (± S.D.). For **f**, **h**, and **i**, statistical significance determined by one-way ANOVA with a Tukey multiple-comparison test. n.s.: not significant; $*p < 0.05$; $**p < 0.01$; $***p < 0.001$; and $****p < 0.0001$. Source data are provided as a Source Data file.
