# Supplementary material for: Proteostasis perturbation of N-Myc leveraging HSP70 mediated protein turnover improves treatment of neuroendocrine prostate cancer
Source: Nat Commun. 2024 Aug 5;15:6626. doi: 10.1038/s41467-024-50459-x (PMC11300456; doi:10.1038/s41467-024-50459-x)
Supplement: Supplementary file 2 — Reporting Summary [file 41467_2024_50459_MOESM2_ESM.pdf]

nature portfolio

Corresponding author(s): Chengfei Liu
Last updated by author(s): 07/03/2024

Reporting Summary

Nature Portfolio wishes to improve the reproducibility of the work that we publish. This form provides structure for consistency and transparency in reporting. For further information on Nature Portfolio policies, see our Editorial Policies and the Editorial Policy Checklist.

Statistics

For all statistical analyses, confirm that the following items are present in the figure legend, table legend, main text, or Methods section.

n/a	Confirmed	
☐	☒	The exact sample size (*n*) for each experimental group/condition, given as a discrete number and unit of measurement
☐	☒	A statement on whether measurements were taken from distinct samples or whether the same sample was measured repeatedly
☐	☒	The statistical test(s) used AND whether they are one- or two-sided<br>*Only common tests should be described solely by name; describe more complex techniques in the Methods section.*
☒	☐	A description of all covariates tested
☒	☐	A description of any assumptions or corrections, such as tests of normality and adjustment for multiple comparisons
☐	☒	A full description of the statistical parameters including central tendency (e.g. means) or other basic estimates (e.g. regression coefficient) AND variation (e.g. standard deviation) or associated estimates of uncertainty (e.g. confidence intervals)
☐	☒	For null hypothesis testing, the test statistic (e.g. *F*, *t*, *r*) with confidence intervals, effect sizes, degrees of freedom and *P* value noted<br>*Give P values as exact values whenever suitable.*
☒	☐	For Bayesian analysis, information on the choice of priors and Markov chain Monte Carlo settings
☒	☐	For hierarchical and complex designs, identification of the appropriate level for tests and full reporting of outcomes
☒	☐	Estimates of effect sizes (e.g. Cohen's *d*, Pearson's *r*), indicating how they were calculated

Our web collection on statistics for biologists contains articles on many of the points above.

Software and code

Policy information about availability of computer code

Data collection	Bio-rad Image Lab version 6.1.0 to acquire the data from Western blots; CFX Maestro Sofware 2.3 to acquire data for RT-PCR
Data analysis	DESeq2, ggplot2, ImageJ, Snapgene, Graphpad Prism 9, SRPLOT, GSEA 4.3.2, R 4.3.1

For manuscripts utilizing custom algorithms or software that are central to the research but not yet described in published literature, software must be made available to editors and reviewers. We strongly encourage code deposition in a community repository (e.g. GitHub). See the Nature Portfolio guidelines for submitting code & software for further information.

Data

Policy information about availability of data

All manuscripts must include a data availability statement. This statement should provide the following information, where applicable:

- Accession codes, unique identifiers, or web links for publicly available datasets
- A description of any restrictions on data availability
- For clinical datasets or third party data, please ensure that the statement adheres to our policy

The RNA-seq data generated from the current study have been deposited in Gene Expression Omnibus (GEO) database with the accession number GSE249917 for UCDCaP and UCDCaP-CR https://www.ncbi.nlm.nih.gov/geo/query/acc.cgi?acc=GSE249917, and GSE249916 for H660 https://www.ncbi.nlm.nih.gov/geo/query/acc.cgi?acc=GSE249916.
The WES data are available by the BioProject ID number (PRJNA1050656). https://dataview.ncbi.nlm.nih.gov/object/PRJNA1050656?

reviewer=nfnli65265imh4q1sii8lc93c
Proteomics data are available at https://massive.ucsd.edu/ProteoSAFe/private-dataset.jsp?task=fbb023432d854b8c93209cd5d93c5d2b.

Research involving human participants, their data, or biological material

Policy information about studies with human participants or human data. See also policy information about sex, gender (identity/presentation), and sexual orientation and race, ethnicity and racism.

Reporting on sex and gender	N/A
Reporting on race, ethnicity, or other socially relevant groupings	N/A
Population characteristics	N/A
Recruitment	N/A
Ethics oversight	N/A

Note that full information on the approval of the study protocol must also be provided in the manuscript.

Field-specific reporting

Please select the one below that is the best fit for your research. If you are not sure, read the appropriate sections before making your selection.

[x] Life sciences   [ ] Behavioural & social sciences   [ ] Ecological, evolutionary & environmental sciences

For a reference copy of the document with all sections, see nature.com/documents/nr-reporting-summary-flat.pdf

Life sciences study design

All studies must disclose on these points even when the disclosure is negative.

Sample size	Sample sizes were chosen based on our experience with the assays performed. Sample sizes were stated in the figure legends.
Data exclusions	No data were excluded.
Replication	For the RT-qPCR assays, three replicate was performed. RNA-seq was generated from three or two replicate as indicated in the manuscript. For cell growth and protein half-life assays, data was shown from three replicate. For the in vivo assay, data was collected and analyzed by the indication in the manuscript. For western blot, two independent technical replicate was performed to ensure the reproducibility. All the results from replicate for each experiment were consistent. Others were indicated in the figure legends.
Randomization	All experiments were based on well established cell lines. The collected cells or lysates were randomly divided into portions for drug treatment or assays. For the in vivo experiment, mice were allocated randomly for acquiring drug treatment
Blinding	There was no blinding for setting up the experiments including drug treatments and manipulation. Processing the collected samples to generate data was blinded such as cell growth and PCR assays.

Reporting for specific materials, systems and methods

We require information from authors about some types of materials, experimental systems and methods used in many studies. Here, indicate whether each material, system or method listed is relevant to your study. If you are not sure if a list item applies to your research, read the appropriate section before selecting a response.

Materials & experimental systems

n/a	Involved in the study	
[ ]	[x]	Antibodies
[ ]	[x]	Eukaryotic cell lines
[x]	[ ]	Palaeontology and archaeology
[ ]	[x]	Animals and other organisms
[x]	[ ]	Clinical data
[x]	[ ]	Dual use research of concern
[x]	[ ]	Plants

Methods

n/a	Involved in the study	
[x]	[ ]	ChIP-seq
[x]	[ ]	Flow cytometry
[x]	[ ]	MRI-based neuroimaging

Antibodies

Antibodies used	N-Myc (#84406, #51705, 1:1000 dilution for WB, 1:200 for IP, 1:100 for IF, Cell Signaling Technology); c-Myc (#5605, 1:1000, Cell Signaling Technology) ; L-Myc (#76266, 1:1000, Cell Signaling Technology); NSE (#MS-335, 1:1000, NeoMarkers); SYP (#PA5-16417, 1:1000, ThermoFisher); CHGA (sc-393941, 1:1000, Santa Cruz Biotechnology); AR (441, sc-7305, 1:1000, Santa Cruz Biotechnology); AURKA (#14475, 1:1000, Cell Signaling Technology); HSC70 (B-6, sc-7298, 1:1000, Santa Cruz Biotechnology); HSP70 (#4873, 1:1000 for WB, 1:200 for IP, Cell Signaling Technology); HSP90 (#ab13492, 1:1000, Abcam), STUB1 (#2080, 1;1000 for WB, 1:100 for IF, Cell Signaling Technology), FLAG® M2 monoclonal antibody (#F1804, 1:1000 for WB, 1:200 for IP, 1:100 for IF, Sigma-Aldrich); His (#66005, 1:5000 for WB, Proteintech); HA (#3724, 1:1000 for WB, 1:200 for IP, 1:800 for IF, Cell Signaling Technology); Ubiquitin (P4D1 and FL76, 1:1000, Santa Cruz Biotechnology); H3 (#ab1220, 1:1000, Abcam), MAX (#sc-8011, 1:1000 for WB, 1:100 for IF, Santa Cruz Biotechnology); GAPDH (#2118, 1:1000, Cell Signaling Technology), secondary antibodies (W4011 and W4021, 1: 5000, Promega; #5127, 1:2000, Cell Signaling Technology).
Validation	N-Myc (D1V2A) Cell Signaling Technology Cat.: #84406, RRID:AB_2800038<br>N-Myc (D4B2Y) Cell Signaling Technology Cat.: #51705, RRID:AB_2799400<br>C-Myc Cell Signaling Technology Cat.: #5605, RRID:AB_1903938<br>L-Myc Cell Signaling Technology Cat.: #76266, RRID:AB_2943075<br>Ki67 (SP6) ThermoFisher Cat. #MA5-14520, RRID:AB_10979488<br>Neuron specific enolase (NSE) NeoMarkers Cat. #MS-335<br>Synaptophysin (SYP) ThermoFisher Cat. #PA5-16417, RRID:AB_10989504<br>chromogranin A (ChgA) Santa Cruz Biotechnology Cat. sc-393941, RRID:AB_2801371<br>androgen receptor (AR) Santa Cruz Biotechnology Cat. sc-7305, RRID:AB_626671<br>Aurora Kinase A (AURKA) Cell Signaling Technology Cat.: #14475, RRID:AB_2665504<br>HSC70 Santa Cruz Biotechnology Cat. sc-7298, RRID:AB_627761<br>HSP70 Cell Signaling Technology Cat.: #4873, RRID:AB_2119694<br>HSP90 Abcam Cat.: ab13492, RRID:AB_300396<br>STUB1 Cell Signaling Technology Cat.: #2080, RRID:AB_2198052<br>FLAG® M2 Sigma Cat.: #F1804, RRID:AB_262044<br>anti-6*His-tag Proteintech Cat.: 66005, RRID:AB_2923721<br>HA-tag Cell Signaling Technology Cat.: #3724, RRID:AB_1549585<br>Ubiquitin (P4D1) Santa Cruz Biotechnology Cat. sc-8017, RRID:AB_628423<br>Ubiquitin (FL 76) Santa Cruz Biotechnology Cat. sc-9133, RRID:AB_2180553<br>Histone H3 Abcam Cat.: ab1220, RRID:AB_449854<br>MAX Santa Cruz Biotechnology Cat. sc-8011, RRID:AB_627913<br>Tubulin Sigma Cat.: #T5168, RRID:AB_477579<br>GAPDH Cell Signaling Technology Cat.: #2118, RRID:AB_561053

Eukaryotic cell lines

Policy information about cell lines and Sex and Gender in Research

Cell line source(s)	LNCaP (ATCC, CRL-2505), C4-2B (a kind gift from Dr. Leland Chung), C4-2B MDVR (C4-2B enzalutamide resistant), PC3 (ATCC, CRL-1435), DU145 (ATCC, HTB-81), and CWR22Rv1 (ATCC, CRL-2505),VCaP (ATCC, CRL-2876), UCDCaP (derived from an 84-year-old male Gleason 10 patient), UCDCaPCR (UCDCaP castration resistant), H660 (ATCC, CRL-5813) and HEK293 (ATCC, CRL-1573.3)
Authentication	Cell types were authenticated using short tandem repeat (STR) profiling, morphology and/or western blot.
Mycoplasma contamination	All the cell lines were tested negative for mycoplasma contamination during the study
Commonly misidentified lines (See ICLAC register)	N/A

Animals and other research organisms

Policy information about studies involving animals; ARRIVE guidelines recommended for reporting animal research, and Sex and Gender in Research

Laboratory animals	5 weeks old male C.B17/lcrHsd-Prkdc-SCID mice (ENVIGO) and 5-week-old male NOD.Cg-Prkdcscid Il2rgtm1Wjl/SzJ (NSG, Envigo) were used. All mice were housed in 22°C, 55% humidity on average with a 12-h light/12-h dark cycle and access to food and water.
Wild animals	N/A
Reporting on sex	only Male mice were used for prostate cancer studies
Field-collected samples	N/A
Ethics oversight	Mice experiment was approved by the Institutional Animal Care and Use Committee (IACUC) at UC Davis.

Note that full information on the approval of the study protocol must also be provided in the manuscript.

Plants

Seed stocks	N/A
Novel plant genotypes	N/A
Authentication	N/A
