# Supplementary material for: Proteostasis perturbation of N-Myc leveraging HSP70 mediated protein turnover improves treatment of neuroendocrine prostate cancer
Source: Nat Commun. 2024 Aug 5;15:6626. doi: 10.1038/s41467-024-50459-x (PMC11300456; doi:10.1038/s41467-024-50459-x)
Supplement: Supplementary file 3 — Description of Additional Supplementary Files [file 41467_2024_50459_MOESM3_ESM.pdf]

Supplementary Data 1:
Annotation summary results from JG231 treated C4-2B N-Myc cells (whole cell lysates). The down regulated proteins from whole cell lysates were input in the Database
for Annotation, Visualization and Integrated Discovery (DAVID). The significant annotations from Gene Ontology, Functional Annotations, and Pathways were listed. Statistical significance was determined by Fisher's exact test.

Supplementary Data 2:
Annotation summary results from JG231 treated C4-2B N-Myc cells (nuclear lysates). The down regulated proteins from nuclear lysates were input in the Database for Annotation, Visualization and Integrated Discovery (DAVID). The significant annotations from Gene Ontology, Functional Annotations, and Pathways were listed. Statistical significance was determined by Fisher's exact test.

Supplementary Data 3:
Oligonucleotide sequences. The qPCR primers, siRNA sequences, and mutagenesis primers were listed.
